# Consistent Interpretation of Time- and Frequency-Domain Traces of Ion Migration in Perovskite Semiconductors

**DOI:** 10.1021/acsenergylett.4c02446

**Published:** 2024-11-12

**Authors:** Moritz C. Schmidt, Agustin O. Alvarez, Jeroen J. de Boer, Larissa J.M. van de Ven, Bruno Ehrler

**Affiliations:** † AMOLF, Science Park 104, 1098 XG Amsterdam, The Netherlands; ‡ University of Groningen, Nijenborgh 3, 9747 AG Groningen, The Netherlands

## Abstract

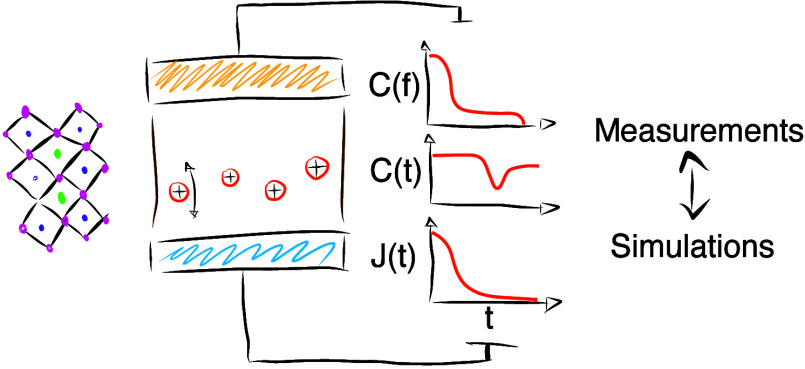

The migration of mobile ions through the metal halide perovskite layer is still one of the main reasons for the poor stability of perovskite solar cells, LEDs, and photodetectors. To characterize mobile ions in the perovskite layer, time- and frequency-based electrical measurements are promising techniques. However, the presence of transport layers complicates their interpretation, limiting the information about mobile ions that can be extracted, and it is not clear how different features in frequency- and time-domain measurements relate to mobile ions. Here, we characterize a transport-layer-free device with capacitance frequency, capacitance transient, and current transient measurements in the dark, under illumination, and at different temperatures. We extract characteristic ionic signatures from the measurements, which we reproduce with drift-diffusion simulations for each technique. This allows us to explain the origins of the different ionic signatures, advancing our understanding of how electronic characterization techniques can be used to study the properties of mobile ions.

All commercial applications of perovskite semiconductors are currently hampered by their poor intrinsic stability.^1^ Long-term degradation occurs through several mechanisms. For example, thermally induced reactions of the soft perovskite crystal release volatile methylammonium (MA) from MAPbI_3_.^2,3^ Humidity and light introduce the decomposition of the perovskite into photoinactive PbI_2_.^4^ Some of these degradation pathways can be avoided by encapsulation, reducing the evaporation of volatile species and the intrusion of moisture.^5,6^ Additionally, perovskite layers exhibit large densities of mobile ions,^7,8^ which dominate the potential distribution within perovskite devices. Recently, these mobile ions have been shown to be the leading cause of the reduction of current extraction of perovskite solar cells, introducing significant efficiency losses on short time scales.^9^ Other perovskite-based devices, such as light-emitting diodes (LEDs) and photodetectors, also suffer from ion-induced degradation.^10−12^ However, mobile ions can also lead to the recovery of perovskite-based devices, also known as self-healing,^13^ and increase the design tolerance for efficient solar cells.^14^ Consequently, reliable ways to quantify mobile ions in perovskite layers are necessary. However, so far, most techniques used to study ion migration have significant downsides. Optical techniques, for example, using photoluminescence spectroscopy to study phase segregation,^15^ only extract quantitative information about the time scale of ion migration and provide no chemical resolution or sensitivity to ions that do not change optical properties. Techniques that offer chemical resolution, such as X-ray fluorescence^16^ and time-of-flight secondary ion mass spectrometry (ToF-SIMS),^17^ require elaborate measurement equipment. Furthermore, X-ray fluorescence requires special sample geometries, usually with larger distances between electrodes, and ToF-SIMS is a destructive technique, not allowing for the study of ion migration while applying a bias. A common way to study ion migration is by electrical measurements, for example, impedance spectroscopy,^18,19^ transient current,^20^ transient voltage,^21,22^ or transient capacitance measurements.^23,24^ While these techniques are often used to extract quantitative information, their interpretation is not trivial, as they are based on complex interactions between ionic and electronic carriers and recombination.^8,25,26^ In addition, it has been shown that transport layers in complete devices complicate the interpretation of many electronic measurements.^26−28^

To circumvent these issues, this work focuses on characterizing the simplest possible perovskite device, an MAPbI_3_ perovskite layer sandwiched between two electrodes without charge transport layers. We compare three different electronic measurements: capacitance frequency (Cf), capacitance transients (Ct), and current transients (Jt) in the dark and under illumination. In most of the measurements, we can identify a signature of an ion migration process. Tracing this feature for various temperatures allowed us to extract its activation energy. With drift-diffusion simulations, we can qualitatively reproduce and explain all measurements when considering a nonradiative recombination process in addition to the mobile ions. While focusing on a single technique to study ion migration often leaves parameters ambiguously defined, using several techniques to measure the same system narrows the parameter set used for the simulations to reproduce the experimental observations. Therefore, we can estimate the mobile ion density, mobility, and activation energy.

We fabricated a simple perovskite device by sandwiching a polycrystalline thin film of MAPbI_3_ between an ITO and a gold electrode (see Figure 1a). The MAPbI_3_ thin film covers the ITO free of pinholes, as shown in the scanning electron microscopy image in Figure S2. Using X-ray diffraction, we can identify all of the characteristic peaks for MAPbI_3_ (see Figure S3). Figure S4a,b shows current density vs voltage (*JV*) measurements at 280, 300, and 320 K in the dark and light. The *JV* measurements are mostly symmetric except for a diode behavior at forward bias. This diode shape is temperature-dependent, and we attribute it to an interfacial barrier. Under illumination, the overall current density of the device increases due to photoconductivity. We chose this device structure to avoid the influence of transport layers on the frequency- and time-dependent electrical measurements. We^26^ and others^27,28^ have previously shown that the transport layers can influence these measurements, complicating their interpretation. The techniques we focus on are the capacitance frequency, capacitance transient, and current transient techniques, as illustrated in Figure 1b. In the capacitance frequency technique, we measure the capacitance with a small voltage perturbation of 20 mV at frequencies ranging from 1 Hz to 500 kHz at 0 V DC voltage. This technique is often applied to perovskite solar cells to study ion migration.^24^ However, it has been shown that the interpretation is difficult, especially when applied to complete devices.^8^ Capacitance transient measurements, originating from deep-level transient spectroscopy,^29^ are less established when characterizing perovskite solar cells. Here, we apply a DC voltage pulse of 1 V and 2.5 s to the ITO (anode). After the voltage pulse, thus at 0 V DC voltage, we measured the change of the capacitance using a high-frequency voltage perturbation of 20 mV and 10 kHz. Even though capacitance transients are difficult to interpret, they contain valuable information about the device properties, as the transients are influenced by the mobility and density of mobile ions.^26^ In the current transient measurements, we apply the same DC voltage pulse of 1 V and 2.5 s to the device and then measure the current as a function of time once the voltage pulse is switched off. This technique has been previously used in complete devices, thin films, and single crystals in attempts to quantify the density of mobile ions.^20,30,31^ We perform all measurements in the dark and under moderate illumination with a 2.3 mW/cm^2^ white light LED. Additional information about the fabrication process and the experimental setup is available in the Experimental Section. To understand the mechanism behind the measurements, we additionally carried out drift-diffusion simulations with the software Setfos by Fluxim. For the ionic parameters, we choose mobile positive ions, accounting for mobile iodide vacancies *V*_I_^+^, and immobile negative ions to conserve charge neutrality (e.g., iodide interstitials *I*_i_^–^ and MA vacancies *V*_MA_^–^).^7,32^ To reproduce the measurements, we choose a work function difference of 0.3 eV between the electrodes, leading to accumulation of mobile positive ions at the anode at steady-state when no voltage is applied. We note that the choice of work function difference affects the steady-state ion distribution, which has a large effect on the simulation results. Furthermore, we additionally included hole traps in the semiconductor layer. We note, however, that we do not know the polarity of the traps in the semiconductor. Therefore, electron traps are also a possibility. Table S1 shows the complete list of the simulation parameters.

**Figure 1:**
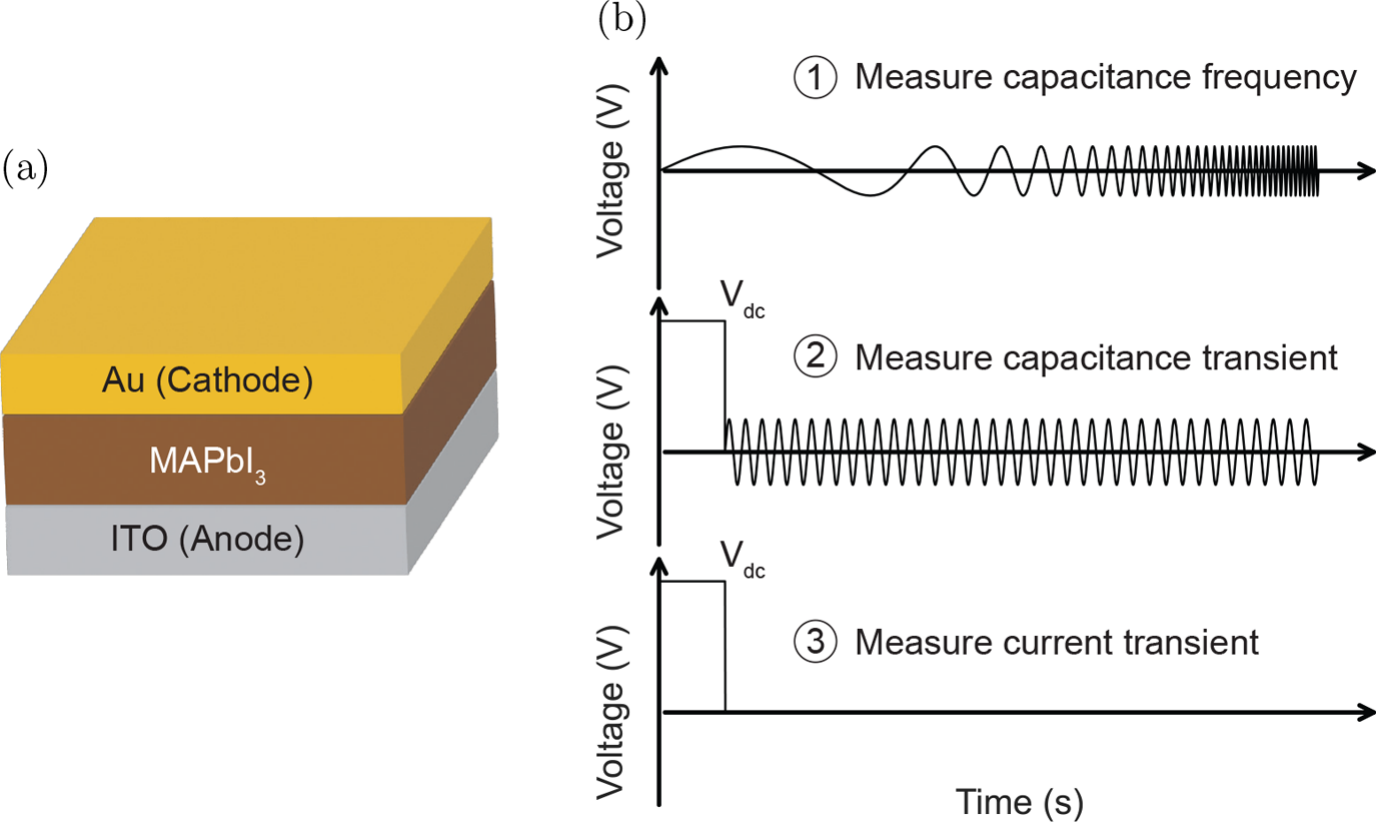
(a) Schematic of the device stack. (b) The three different techniques used in this work: capacitance frequency, capacitance transient, and current transient techniques.

The first technique we focus on is the capacitance frequency technique, where we measure the capacitance *C* of the device at different frequencies *f*. The capacitance can be determined from the impedance *Z*:
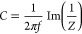
1The measured capacitance in the dark for different temperatures from 280 to 320 K is plotted in Figure 2a. At intermediate frequencies from 50 Hz to 50 kHz, we observe a plateau in the capacitance value. Because no transport layers are present, we can assume that the geometric capacitance dominates the capacitance in this regime. Using the approximation of a parallel plate capacitor
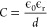
2with ϵ_0_ being the vacuum permittivity and *d* the thickness of the perovskite of 330 nm, we can approximate the permittivity ϵ_r_ of the perovskite to be 54 at 300 K, close to values previously observed in the literature.^33^ At frequencies below 50 Hz, mobile ions in the perovskite start to polarize, increasing the capacitance.^34,35^ We reproduce this capacitance increase at low frequencies using drift-diffusion simulations, as shown in Figure S5a. The drift-diffusion simulations allow us to distinguish between the contributions to the total capacitance *C*_tot_ from electrons *C*_n_, holes *C*_p_, positive ions *C*_ion_, and the time-dependent change of the electric field, i.e., displacement, *C*_disp_. These contributions are shown in Figure 2c for a capacitance frequency simulation in the dark at 300 K. At frequencies smaller than 20 Hz, mobile ions dominate the total capacitance. At higher frequencies, the device’s capacitance is entirely dominated by the displacement current, confirming that an approximation of the geometrical capacitance is valid.

**Figure 2:**
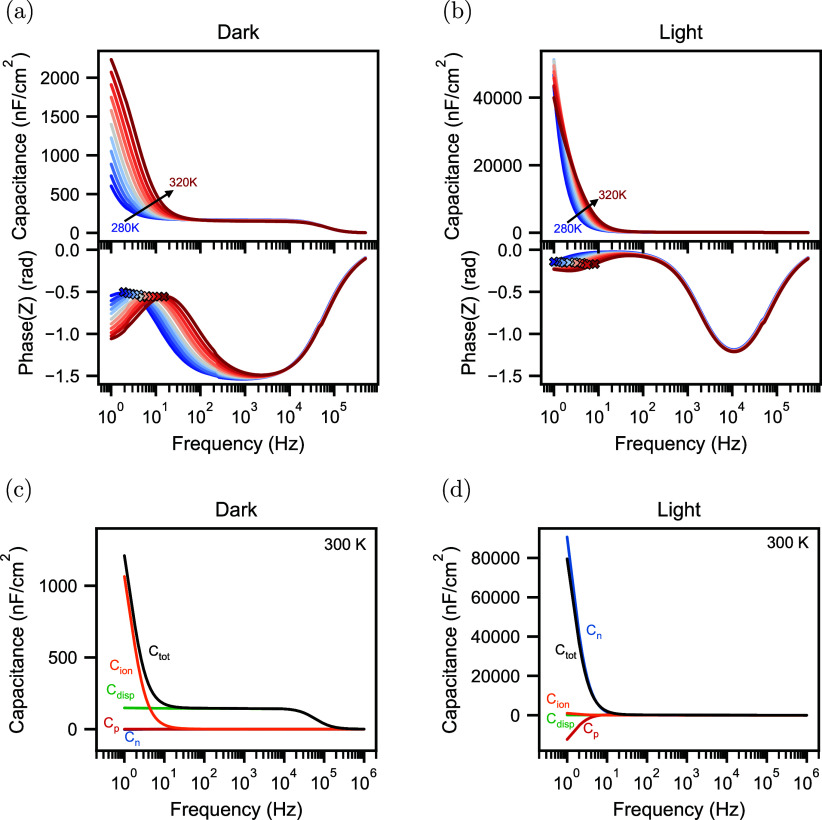
(a) Capacitance frequency measurements and corresponding phase angles in the dark at temperatures from 280 to 320 K in steps of 4 K. (b) Capacitance frequency measurements and corresponding phase angles in light. (c) Simulated contributions of electrons *C*_n_, holes *C*_p_, ions *C*_ion_, and displacement *C*_disp_ to the total capacitance *C*_tot_ for the capacitance frequency simulations in the dark at 300 K. (d) Simulated contributions to the capacitance in light at 300 K.

The characteristic time of the ionic signature can be estimated by the peak of the phase of the impedance. The measurement of the phase angle is shown in Figure 2a, and the simulations are shown in Figure S5a. This characteristic time is inversely proportional to the ionic conductivity:
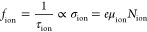
3where σ_ion_ is the ionic conductivity, μ_ion_ is the mobility of ions, *N*_ion_ is the density of mobile ions, and *e* the elementary charge. This relationship between the phase peak and ionic conductivity is illustrated in Figure S6. As the mobility of the ions decreases with temperature,^36^ the characteristic frequency also decreases with decreasing temperature.

Next, we perform the same Cf measurements while illuminating the device with a low-intensity white light LED. Compared to the dark measurements, the capacitance increases around one order of magnitude at low frequencies, as shown in Figure 2b. We can reproduce this large capacitance rise with illumination at different temperatures in drift-diffusion simulations, as shown in Figure S5b. We attribute this to the contribution of electronic carriers in the device in combination with a phase delay due to mobile ions. According to Jacobs et al.,^25^ electronic carriers contribute to the impedance of a device with a charge storage *Z*_Q_ and recombination *Z*_R_ term. Based on the current continuity equation, these contributions for electrons, for example, can be expressed as
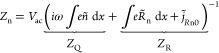
4where *ñ* is the AC electronic carrier density,  is the AC recombination rate of electrons in the bulk, and  is the AC recombination current of photogenerated electrons at the anode. By substituting eq 4 into eq 1, we get the contributions of the charge storage and recombination terms to the capacitance:
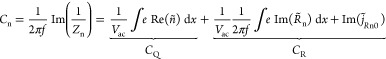
5We want to emphasize that the contribution *C*_R_ is not a capacitance in the classical sense; i.e., it does not describe the device’s ability to store charge. Instead, recombination processes can lead to phase-delayed currents similar to the conventional charge storage capacitance *C*_Q_. Therefore, the capacitances of, for example, electrons *C*_n_ mentioned here only describe the phase delay of the electron current and the applied voltage rather than the device’s ability to store electrons. At low frequencies, mobile ions introduce a phase shift in the potential and the electronic carrier densities, resulting in significant contributions of the recombination term *C*_R_ to the capacitance, which is responsible for the large rise of the capacitance. In other words, the photogenerated carriers recombine, and the phase delay that the mobile ions induce in this recombination process leads to a large increase in capacitance. In the simulations, we again break down the different contributions to the capacitance at 300 K and see that the electron and hole capacitances dominate the total capacitance at low frequencies (Figure 2d). Furthermore, we calculate the contributions of charge storage and recombination to the overall capacitance according to eq 5 (the derivation of the AC recombination is described in the Supporting Information). The result in Figure S7a illustrates that the out-of-phase recombination of electrons and holes, i.e., *C*_R_ in eq 5 for electrons, dominates the capacitance rise at low frequencies. As this out-of-phase recombination is caused by the movement of ions, we can still estimate a characteristic time, for example, by choosing the inflection point between the low-frequency dip and peak, as illustrated in Figure 2b and reproduced with simulations in Figure S5b. We note that we would ideally choose the minimum of the phase as the characteristic time, as it correlates with the characteristic time of the low-frequency semicircle in the Nyquist plot of the impedance. However, we cannot resolve this characteristic point at low temperatures because it is too slow to be measured within a reasonable time. This effect is also illustrated in the Nyquist plots in Figure S8, where only a small fraction of the low-frequency arc is visible at low temperatures (280 K). It is noteworthy that the observed large increase of the capacitance at low frequency is not limited to devices with perovskite/metal interfaces, i.e., devices with high interfacial recombination. It is commonly observed in high-performing perovskite solar cells and can generally be attributed to phase-delayed recombination due to mobile ions.^25,26^

Next, we focus on the impact of mobile ions on capacitance transient measurements. In these, we measure the capacitance after applying a voltage pulse to the device. During the voltage pulse, mobile ions drift and accumulate at the perovskite/cathode interface. After the pulse, mobile ions drift back into the perovskite bulk, and we measure the capacitance. Figure 3a shows the transients measured in the dark. While the capacitance decreases with increasing temperature, the transients do not show significant changes over time. We attribute this to the fact that we mainly probe the geometrical capacitance at 10 kHz, which is not significantly modulated when ionic carriers are redistributed within the device. In simulations, we only see a slight modulation of the capacitance in the dark (see Figure S5c), which might be hidden by noise in the experiment. This minor change of the capacitance originates from a modulation of *C*_disp_, as illustrated in Figure 3c. The observed temperature shift could originate from a temperature-dependent dielectric constant of the perovskite, as similarly observed in the literature.^37^ In contrast to the device studied here, significant dynamics are usually observed when measuring capacitance transients of complete solar cells in the dark,^23,24^ which we mainly attribute to the modulation of the transport layer capacitances in our previous work.^26^ As no charge transport layers are present in the devices studied here, the measured transients do not show any dynamics.

**Figure 3:**
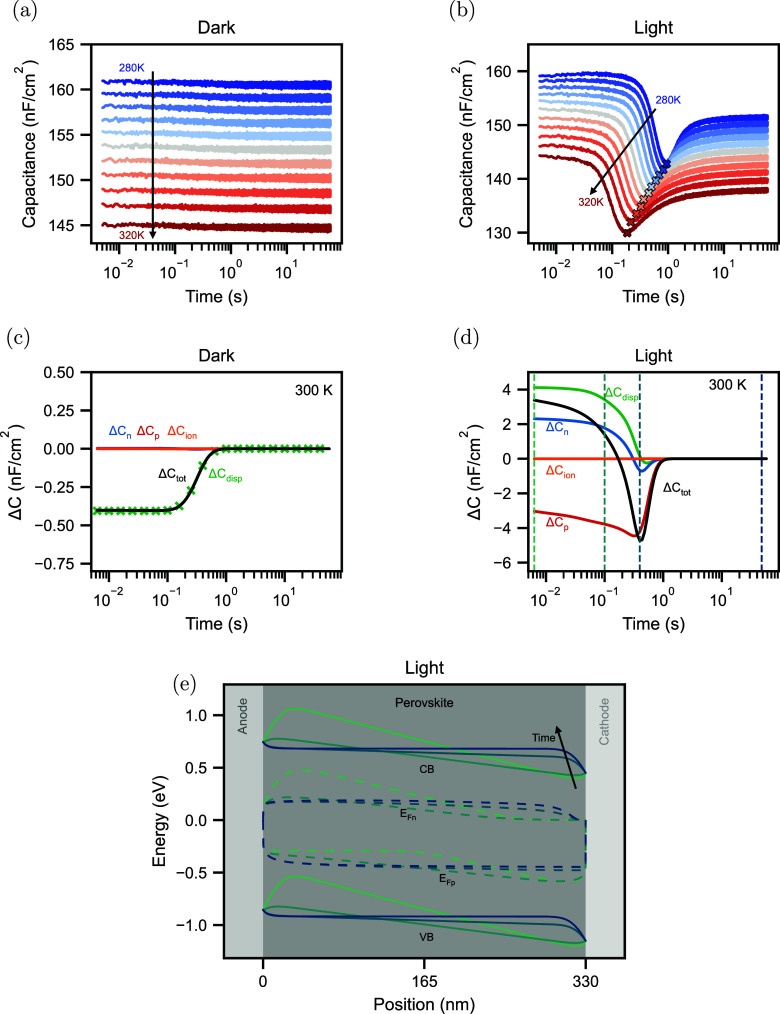
(a) Capacitance transient measurements in the dark at temperatures from 280 to 320 K in steps of 4 K. (b) Capacitance transient measurements in light. (c) Simulated contributions of electrons Δ*C*_n_, holes Δ*C*_p_, ions Δ*C*_ion_, and displacement Δ*C*_disp_ to the total capacitance Δ*C*_tot_ for the capacitance transient simulations in the dark at 300 K. (d) Simulated contributions to the total capacitance for the capacitance transient simulations in light at 300 K. Different points in time are marked with vertical dashed lines. (e) Band diagram under illumination at these times.

Interestingly, we observed significant dynamics when measuring capacitance transients while illuminating the device, as shown in Figure 3b. The capacitance first decreases, followed by a rise before it stabilizes. The dip is strongly temperature-dependent, shifting from around 1 s at 280 K to 0.2 s at 320 K. We carry out drift-diffusion simulations of the capacitance transients with the same device parameters used in the capacitance frequency simulations. Initially, the mobile ions are accumulated at the cathode. Then, after removing the voltage pulse, the ions drift away from the cathode and accumulate at the anode (see Figure S9). Simulating the capacitance transient during the redistribution of ions reproduces the temperature-dependent dip of the capacitance, as shown in Figure S5d. We can again distinguish between the different contributions to the capacitance or, more specifically, the modulation of the capacitance Δ*C*. These are illustrated in Figure 3d for 300 K. The capacitance decrease originates mainly from a decrease in *C*_disp_ and *C*_n_, whereas the rise is dominated almost entirely by *C*_p_. The contributions of *C*_n_ and *C*_p_ are again a result of the out-of-phase recombination, as Figure S7b clarifies. In contrast to the low-frequency capacitance, however, the out-of-phase recombination at 10 kHz is not impacted by the slow response of mobile ionic carriers to the AC perturbation. Instead, it mainly depends on the AC recombination dynamics at the electrode and in the bulk due to electronic trap states. These AC recombination dynamics depend on a complex interplay among the electronic carrier distributions, capture coefficients, and frequency of the applied AC potential. This can be seen in eqs S10 and S11 for the AC recombination rates of holes and electrons. When, for example, the trap capture rates of electrons *c*_n_ or holes *c*_p_ are varied, the amplitude of the rise of the capacitance changes (see Figure S10a,b). The probing frequency also impacts the rise of the capacitance, as illustrated in Figure S10c. Even though the recombination rates are not directly dependent on mobile ions, they are indirectly dependent, as the distribution of mobile ions impacts the band profile. This is illustrated in Figure 3e for four specific times marked with dashed lines in Figure 3d. Initially, when most ions are still accumulated at the cathode, the valence band at the anode and the conduction band at the cathode side have a high electronic carrier density. Then, as mobile ions drift away from the cathode and redistribute, the conduction and valence bands are flatter and populated more homogeneously across the perovskite. This change in the electronic carrier distribution across the perovskite ultimately impacts the AC recombination, resulting in the dip of the capacitance transients. Interestingly, we have observed a dependency of the capacitance rise on illumination intensity in full perovskite solar cells in our previous work,^26^ which we attributed to the modulation of the charge storage capacitance due to mobile ions. However, based on the results presented here, we hypothesize that the modulation of phase-delayed recombination can also impact the capacitance transients of complete devices.

Finally, we carried out current transient measurements. The results in the dark are shown in Figure 4a. Here, we measure the current density after applying the same voltage pulse as that in the capacitance transient measurements. Mobile ions are first accumulated at the cathode and then drift into the perovskite bulk, leading to a compensation current on the electrodes that can be extracted from the device. With drift-diffusion simulations, we can reproduce this behavior (see Figure 4c) and observe that the entire current is dominated by the ionic current *J*_ion_. A decrease in the amplitude with lower temperatures is observed in Figure 4a. This trend can be explained by the lower mobility of mobile ionic carriers at lower temperatures and can also be seen in the corresponding temperature-dependent simulations in Figure S5e. To extract a characteristic time of the ionic signature, we fit a stretched exponential to the transients (see the Supporting Information for more details) and extract the amplitude *J*_0_ and the characteristic times τ of the transients, which are shown in Figure 5a.

**Figure 4:**
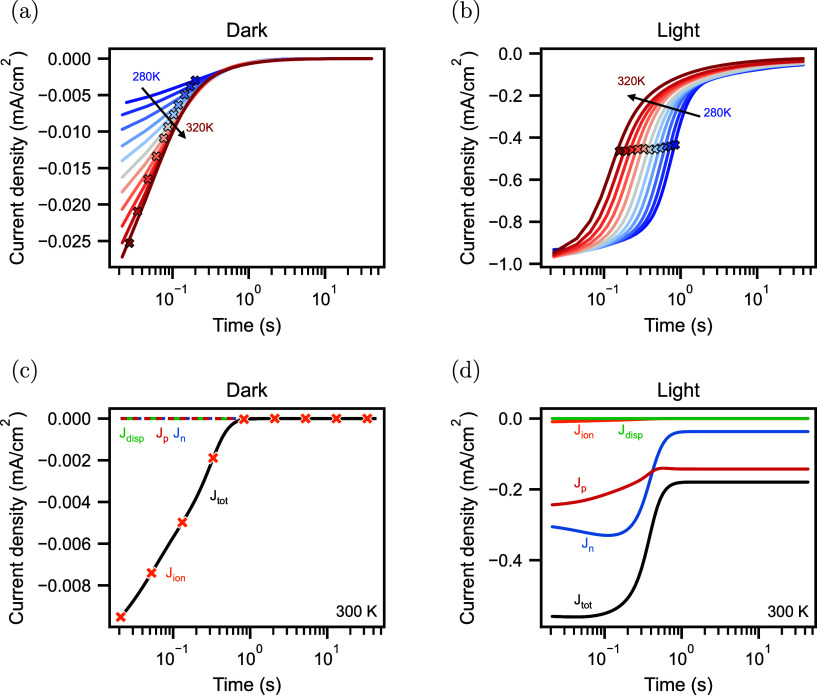
(a) Current transient measurements in the dark at temperatures from 280 to 320 K in steps of 4 K. (b) Current transient measurements in light. (c) Simulated contributions of electrons *J*_n_, holes *J*_p_, ions *J*_ion_, and displacement *J*_disp_ to the total current *J*_tot_ for the current transient simulations in the dark at 300 K. (d) Simulated contributions to the total current for the current transient simulations in light at 300 K.

**Figure 5:**
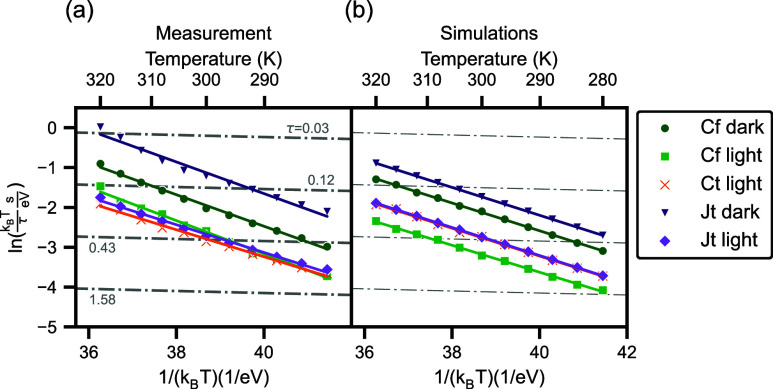
(a) Characteristic times of the ionic signatures at various temperatures of capacitance frequency (Cf), capacitance transient (Ct), and current transient (Jt) measurements. (b) Characteristic times of the simulations shown in Figure S5. The lines represent exponential fits accounting for the temperature-activated diffusion coefficient of the mobile ions to extract their activation energies listed in Table 1.

The current transients under illumination are shown in Figure 4b. Here, the current is not dominated by mobile ions, in contrast to the dark measurements. Instead, we measured the extraction of photogenerated carriers from the device. After the voltage pulse, mobile ions accumulate at the cathode and are depleted at the anode, as illustrated in Figure S9. This leads to a nonzero electric field in the perovskite bulk and, consequently, the extraction of electronic carriers. However, when the mobile ions redistribute, the electric field in the bulk vanishes (the conduction and valence bands in the bulk are flat at later times; see Figure 3e), and the recombination of photogenerated carriers in the bulk increases. This reduces the extracted electron and hole currents, as shown in the simulations in Figure 4d. Notably, the total currents of the simulations in Figure 4d and Figure S5f do not match the absolute values of the measurements. We attribute this discrepancy to differences in the trap distribution in the measurements and simulations. From the measurements, we extract a characteristic time similar to the dark current transient measurements by fitting a stretched exponential to the transients.

Now, we have qualitatively understood the origin of the different ionic signatures of the different measurements, and we can compare their characteristic times quantitatively. The extracted time constants are shown in Figure 5. Assuming a temperature-activated diffusion coefficient of the mobile ionic carriers^36,38^
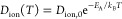
6where *k*_B_ is the Boltzmann constant and *T* is the temperature, we extract the activation energies *E*_A_ for each technique, listed in Table 1. For the capacitance frequency measurements in the dark and light, we extract activation energies of 0.40 and 0.42 eV, respectively. For the current transients, we extract 0.40 eV for the dark measurements and 0.35 eV for the light measurements from fitting the time constants. We additionally used the fitted amplitude of the current transients to determine the activation energy to be 0.40 eV in the dark, as shown in Figure S11. Lastly, we extract 0.34 eV for the capacitance transient measurement under illumination. When the extracted characteristic times for the different techniques are compared, it is apparent that the ones measured under illumination are similar. The characteristic time of the impedance measurement in the dark is lower, followed by the characteristic time of the current transient measurements in the dark. We can roughly reproduce this trend when extracting the same characteristic time constants from simulated data, assuming an activation energy of 0.35 eV, as depicted in Figure 5b. The capacitance transient and current transient simulations under illumination have the same time constants. In contrast, the extracted time constants of the capacitance frequency simulations under illumination are slightly higher. We attribute this to the fact that the low-frequency response of the capacitance frequency is heavily influenced by the recombination dynamics in the device, leaving more parameters to influence the characteristic time. This also becomes clear in the extracted activation energy for the capacitance frequency simulation under illumination, which is 0.01 eV off from the set value of 0.35 eV. In contrast, the extracted activation energies of the other techniques are accurate. Lastly, the extracted time constants of the two dark methods, capacitance frequency and current transient, lie at lower values than the light simulations, following the same trend observed in the measurements.

**Table 1:** Extracted Activation Energies of the Different Techniques Capacitance Frequency (Cf), Capacitance Transient (Ct), and Current Transient (Jt) in the Dark and Light^a^

	activation energy (eV)	
technique	measurement	simulation
Cf dark	0.40 ± 0.01	0.35
Cf light	0.42 ± 0.01	0.34
Ct light	0.34 ± 0.01	0.35
Jt dark	0.40 ± 0.02	0.35
Jt light	0.35 ± 0.01	0.35

a The error corresponds to the error of the fit. If a value does not have an error, the error of the fit is lower than the least significant digit. The corresponding fits to extract the activation energies are shown in Figure 5.

Because we can reproduce all the different features observed in the different methods in the dark and under illumination and the extracted characteristic times follow a similar trend, we can estimate the mobile ion density for the MAPbI_3_ thin film to be around the values used for the drift-diffusion simulation, i.e., 2 × 10^18^ cm^–3^, and a diffusion coefficient of 3.96 × 10^–11^ cm^2^/s at 300 K. Lastly, we estimate the activation energy as the average of the different measurements to be around 0.38 ± 0.03 eV.

In this work, we have successfully combined three different measurements, namely, capacitance frequency, capacitance transient, and current transient measurements, to characterize mobile ions in a simple ITO/MAPbI_3_/Au device. By choosing this simple structure, we could avoid the impact of transport layers, which often complicate the characterization of mobile ions. We measured all techniques at various temperatures, both in the dark and under low illumination conditions. We were able to identify ionic signatures in all measurements except for the dark capacitance transients. Using drift-diffusion simulations, we offered explanations for the origins of the different ionic features. In the dark, the observed features originate directly from the ionic carriers within the device. In contrast, the features in the light measurements are dominated by the modulation of recombination dynamics due to mobile ions. More specifically, the mobile ion distribution within the device directly impacts the electronic carrier distribution and, in turn, the recombination dynamics, ultimately resulting in observable ionic features in the different measurements. These findings underline the importance of taking mobile ions into account when characterizing recombination dynamics of perovskite semiconductors. Lastly, we extracted and compared the characteristic time constants of the different ionic features in the measurements and the simulations. This allowed us to estimate the mobile ion density, diffusion coefficient, and activation energy in the MAPbI_3_ semiconductor.

## Experimental Section

### Device Fabrication

#### Materials

Acetone (anhydrous, ≥99.8%) and isopropanol (IPA, anhydrous, ≥99.8%) were purchased from Biosolve. PbI_2_ (99.99%) was purchased from TCI. Methylammonium iodide (MAI, purity not listed) was purchased from Solaronix. *N*,*N*-Dimethylamine (DMF, anhydrous, ≥99.8%) and chlorobenzene (anhydrous, ≥99.8%) were purchased from Sigma-Aldrich. All materials were used without further purification.

#### Substrates

Patterned quartz/ITO substrates were cleaned by scrubbing with water and soap, followed by sequential sonication in deionized water, acetone, and IPA (15 min per liquid).

#### Perovskite Layer

All processing steps were performed in a nitrogen-filled glovebox. For the precursor solution, 322.8 mg of MAI was dissolved in 2 mL of dimethylformamide in a vial. The vial was stirred vigorously, and the clear, colorless liquid was added to another vial containing 936.0 mg of PbI_2_. The solution was then stirred overnight (400 rpm, 50 °C) to ensure the complete dissolution of the powders. The MAPbI_3_ precursor solution was then filtered with a 0.2 μm PTFE filter. The resulting bright yellow mixture was spin-coated using a spin-coating robot (Sciprios SpinBot, 4000 rpm, 30 s, 4000 rpm/s acceleration) on the quartz/ITO substrates. As an antisolvent, 250 μL of chlorobenzene was added to the spinning substrates after 5 s of spinning. The substrates were annealed (100 °C for 10 min) immediately after spin-coating, resulting in MAPbI_3_ perovskite thin films.

#### Top Electrode

For the top electrode, a thermal evaporator was used to evaporate 100 nm of gold through a shadow mask at a pressure of 1 × 10^–6^ mbar onto the perovskite layer.

### Electrical Characterization

All electrical measurements were carried out inside a Janis VPF-100 liquid nitrogen cryostat. During the measurements, the pressure in the cryostat was below 5 × 10^–6^ mbar. The temperature was controlled by using a Lakeshore 335 temperature controller and was stabilized for 10 min before each measurement cycle. We used a white light LED SOLIS-3C by Thorlabs for measurements with illumination. The capacitance frequency measurements were carried out using an MFIA instrument by Zurich Instruments with an AC voltage perturbation of 20 mV and by sweeping the frequency from 1 Hz to 500 kHz. The capacitance transient measurements were carried out with the same voltage perturbation of 20 mV at a constant frequency of 10 kHz. For the voltage pulse, 1 V was applied to the ITO electrode for 2.5 s. The current transient measurements were acquired using an Agilent B2902A instrument. Here, we also applied a voltage pulse of 1 V for 2.5 s. The *JV* measurements were acquired with an Agilent B2902A instrument from −1 to 1 V with a scan speed of 0.5 V/s.

### Thin Film Characterization

Scanning electron microscopy (SEM) images of the perovskite devices on ITO were recorded in a vacuum on an FEI Verios 460 instrument. The accelerating voltage used was 5 kV, and a 100 pA current was used. X-ray diffraction (XRD) patterns of the perovskite solar cell devices on ITO were recorded on a Bruker D2 Phaser instrument with Cu Kα X-rays with λ = 1.54 Å as the X-ray source. A 0.1 s exposure time, 0.6 mm slit width, 1 mm knife height, and 0.016° (2θ) step size were used.

## Supporting Information Available

Derivation of the AC recombination constants, supporting figures, and the drift-diffusion simulation parameters (PDF)
