# Supplementary material for: Consistent Interpretation of Time- and Frequency-Domain Traces of Ion Migration in Perovskite Semiconductors
Source: ACS Energy Lett. 2024 Nov 12;9(12):5850–8. doi: 10.1021/acsenergylett.4c02446 (PMC11650776; doi:10.1021/acsenergylett.4c02446)
Supplement: Supplementary file 1 — nz4c02446_si_001.pdf [file nz4c02446_si_001.pdf]

Supporting information to Consistent interpretation of time- and frequency-domain traces of ion migration in perovskite semiconductors

Moritz C. Schmidt,[†] Agustin O. Alvarez,[†] Jeroen J. de Boer,[†] Larissa J.M. van de Ven,[†,‡] and Bruno Ehrler[*,†,‡]

†*AMOLF, Science Park 104, 1098 XG, Amsterdam, The Netherlands*

‡*University of Groningen, Nijenborgh 3, 9747 AG, Groningen, The Netherlands*

E-mail: b.ehrler@amolf.nl

Fitting of current transients

To extract characteristic times from the current transient measurements, we fit the decays with stretched exponential decays:

$$J(t) = J_0 e^{-(\frac{t}{\tau})^\alpha} + J_{offset}$$

We chose to fit the transients with a stretched exponential because the quality of fits was better compared to single exponential fits. The stretched exponential function could indicate that a distribution of timescales describes the long-time decay most accurately.[1] Figure S1 shows an example fit of the current transient in the dark at 300 K.

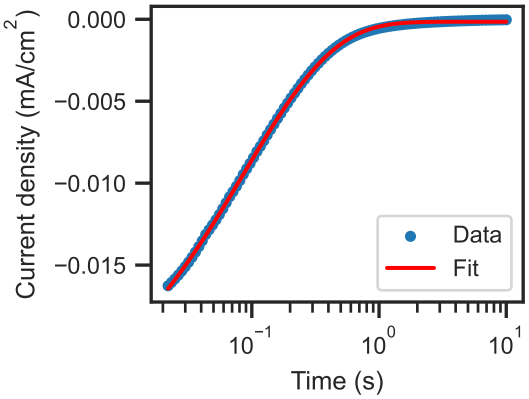

Figure S1: Example fit of a current transient measured in the dark at 300 K

Derivation of AC recombination

We can derive the AC recombination constants of electrons $\tilde{R}_n$ and holes $\tilde{R}_p$ assuming hole traps similar to literature.[2] We start with the DC recombination rates R_p and R_n:[3]

$$R_p = c_p p(N_t - p_t) - e_p p_t \tag{S1}$$

$$R_n = c_n n p_t - e_n(N_t - p_t) \tag{S2}$$

where c_n and c_p are the capture coefficients for electrons and holes, n and p are the electron and hole densities, N_t is the trap density, p_t is the density of trapped holes, and e_n and e_p are the electron and hole emission rates. The rate of change of trapped holes is:

$$\frac{dp_t}{dt} = R_p - R_n \tag{S3}$$

We then extend the different variables into DC and AC parts, for example for holes by:

$$p = p_0 + \tilde{p}e^{i\omega t} \tag{S4}$$

If we assume that emission is negligible compared to recombination (as is the case for deep traps in our simulations), we can omit the emission terms in Equations S1 and S2. Together with Equation S3 this results in:

$$i\omega\tilde{p}_t e^{i\omega t} = c_p(p_0 + \tilde{p}e^{i\omega t})(N_t - (p_{t0} + \tilde{p}_t e^{i\omega t})) - c_n(n_0 + \tilde{n}e^{i\omega t})(p_{t0} + \tilde{p}_t e^{i\omega t}) \tag{S5}$$

We divide this into an DC and an AC part, where we omit the terms of higher order frequency:

$$0 = c_p p_0(N_t - p_{t0}) - c_n n_0 p_{t0} \tag{S6}$$

$$i\omega\tilde{p}_t = c_p(\tilde{p}(N_t - p_{t0}) - p_0\tilde{p}_t) - c_n(n_0\tilde{p}_t + \tilde{n}p_{t0}) \tag{S7}$$

Solving for p_{t0} and $\tilde{p}_t$ we get:

$$p_{t0} = \frac{N_t c_p p_0}{c_n n_0 + c_p p_0} \tag{S8}$$

$$\tilde{p}_t = \frac{c_p\tilde{p}(N_t - p_{t0}) - c_n\tilde{n}p_{t0}}{i\omega + c_p p_0 + c_n n_0} \tag{S9}$$

Substituting Equation S9 into the AC extension of Equation S1 and S2, we finally get an expression for the AC recombination rate for holes and electrons:

$$\begin{aligned}\tilde{R}_p &= c_p(\tilde{p}(N_t - p_{t0}) - p_0\tilde{p}_t) \\ &= c_p c_n \frac{\tilde{p}(N_t - p_{t0})(n_0 + i\omega/c_n) + \tilde{n}p_{t0}p_0}{i\omega + c_p p_0 + c_n n_0}\end{aligned} \tag{S10}$$

$$\begin{aligned}\tilde{R}_n &= c_n n_0 \tilde{p}_t + c_n \tilde{n} p_{t0} \\ &= c_n c_p \frac{n_0\tilde{p}(N_t - p_{t0}) + \tilde{n}p_{t0}(p_0 + i\omega/c_p)}{i\omega + c_p p_0 + c_n n_0}\end{aligned} \tag{S11}$$

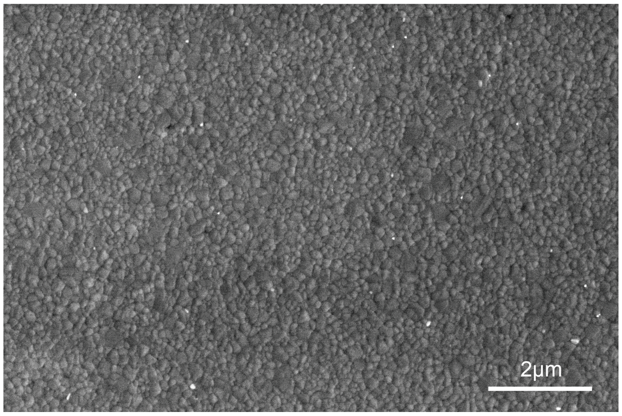

Figure S2: Scanning electron microscopy image of a $MAPbI_3$ thin film on ITO.

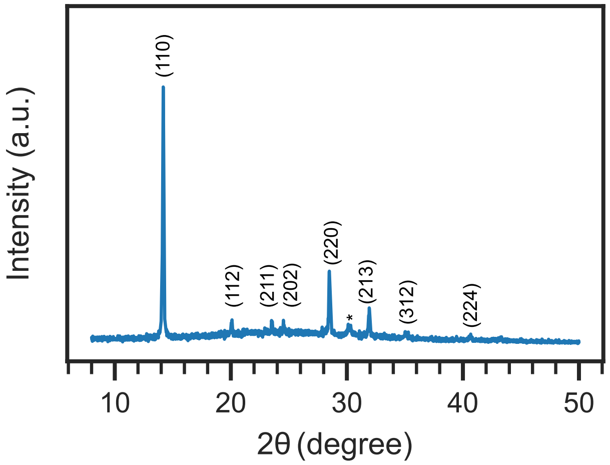

Figure S3: X-ray diffraction pattern of the $MAPbI_3$ thin film on ITO. The crystal planes were assigned by comparing the 2θ angles to literature.[7] The peak at 30° is due to the ITO.

Table S1: Parameters used for the drift-diffusion simulations.

**Parameter**	**Value**	**Comment**
Band gap perovskite (eV)	1.6	From [4]
Electron affinity (eV)	3.9	From [4]
Dielectric constant ϵ_r	54	Estimated from capacitance at 10 kHz and 300 K
Thickness perovskite (nm)	330	Measured with profilometer
Effective density of states conduction band $N_{0,CB}$ (cm^{-3})	$8 \cdot 10^{18}$	
Effective density of states valence band $N_{0,VB}$ (cm^{-3})	$8 \cdot 10^{18}$	
Bimolecular recombination coefficient (cm^{-3}/s)	$1 \cdot 10^{-11}$	
Hole trap density N_t (cm^{-3})	$9 \cdot 10^{15}$	
Capture rate electrons c_n (cm^3/s)	$5 \cdot 10^{-7}$	
Capture rate holes c_p (cm^3/s)	$5 \cdot 10^{-7}$	
Trap energy with respect to VB E_t (eV)	0.7	
Mobility electrons μ_n (cm^2/Vs)	11	In the range of [5,6]
Mobility holes μ_p (cm^2/Vs)	11	In the range of [5,6]
Mobile positive ion density N_{ion} (cm^{-3})	$2 \cdot 10^{18}$	
Immobile negative ion density N_{nion} (cm^{-3})	$2 \cdot 10^{18}$	
Diffusion coefficient of ions at 300 K $D_{ion,300K}$ (cm^2/s)	$3.96 \cdot 10^{-11}$	
Activation energy of diffusion coefficient of ions $E_{a,ion}$ (eV)	0.35	
Anode work function $W_{f,anode}$ (eV)	4.65	
Cathode work function $W_{f,cathode}$ (eV)	4.35	
Series resistance R_s (Ωcm^2)	18.375	

(a) (b)

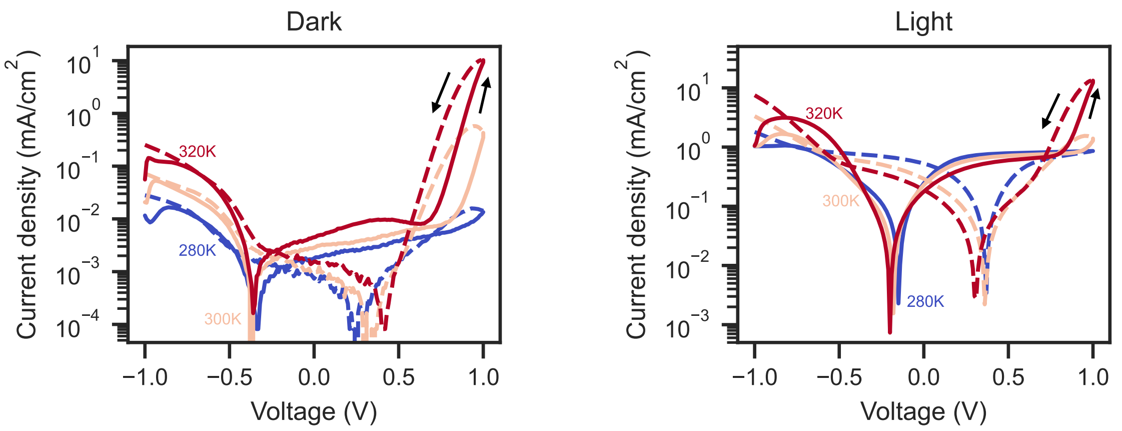

Figure S4: Current-density vs. voltage measurements of a ITO/$MAPbI_3$/Au device in (a) dark and (b) light (2.3 mW/cm^2) at three different temperatures. The measurements were carried out with a scan speed of 0.5 V/s from −1 to 1 V. The arrows indicate the measurement direction.

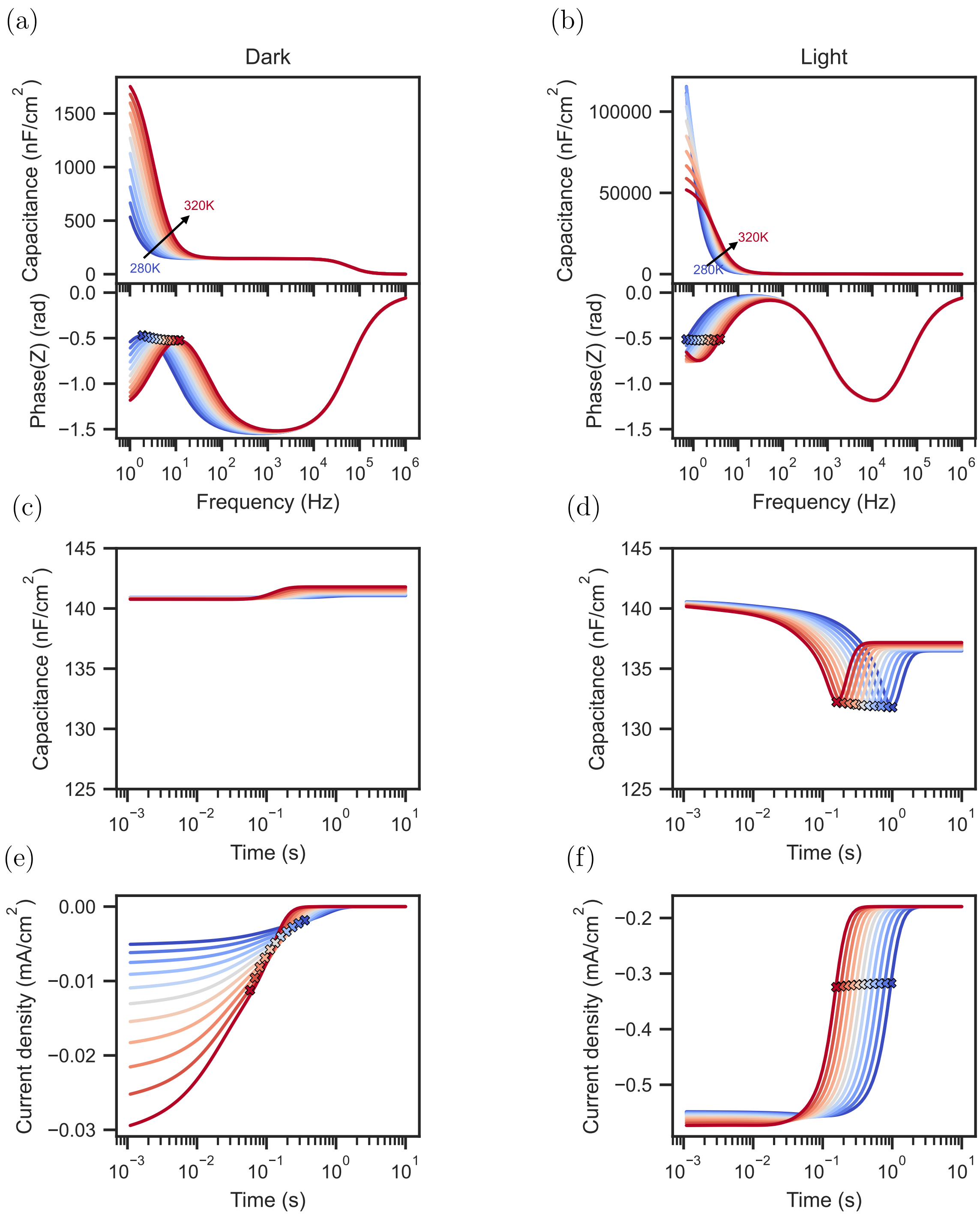

Figure S5: Different simulations of a perovskite semiconductor at temperatures from 280-320 K in steps of 4 K, using the parameters in Table S1. All figures in the left column contain simulations in the dark. The right column contains simulations under illumination with a white LED at an irradiance of $2.3\,\mathrm{mW/cm^2}$. (a) Capacitance frequency simulations in the dark and (b) in the light. (a) Phase(Z) simulations in the dark and (b) under illumination. (c) Capacitance transient simulations in the dark and (d) in light. (e) Current transient simulations in the dark and (f) in light.

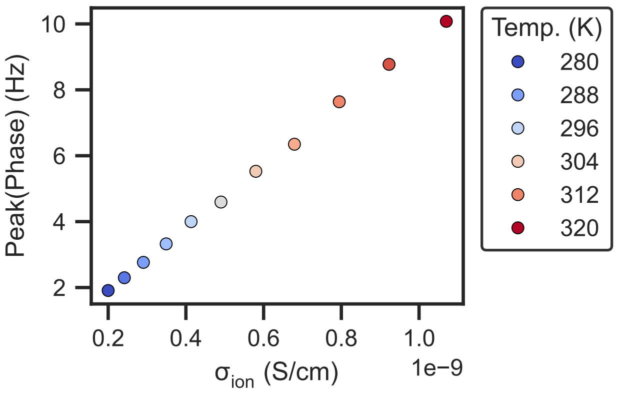

Figure S6: Illustration of the proportionality between the characteristic time of the phase peak of the Cf simulations in the dark of Figure S5(a) and the ionic conductivity of the perovskite layer.

(a) (b)

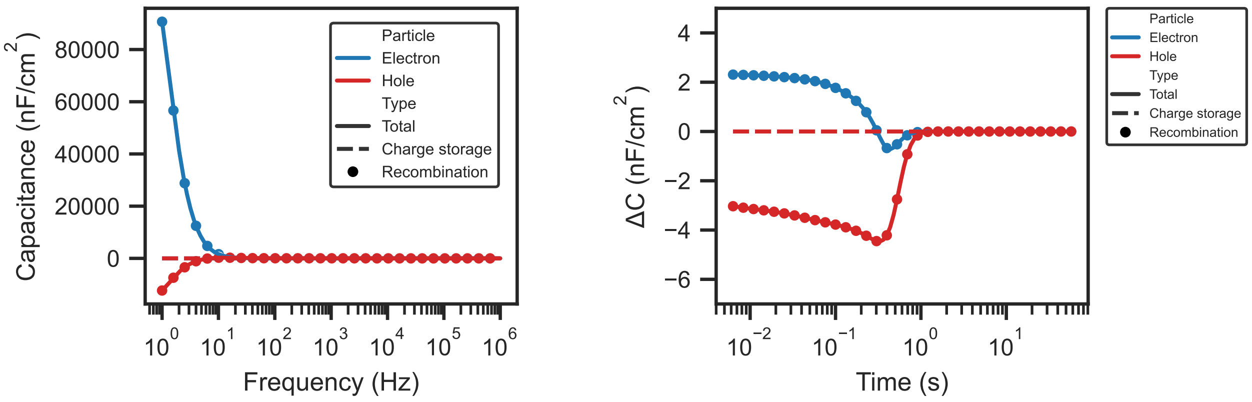

Figure S7: Contributions of the charge storage and recombination of electrons and holes to the capacitance for (a) the capacitance frequency simulations under illumination shown in 2(d) and (b) capacitance transient simulations under illumination in 3(d).

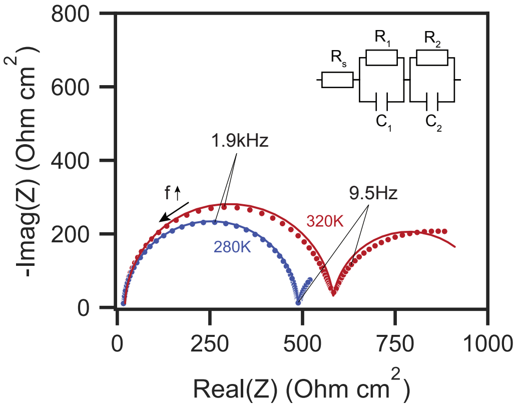

Figure S8: Nyquist plots of the impedance measurements of the device under illumination at 280 K and 320 K. The lines correspond to fits with the illustrated equivalent circuit. At 280 K the majority of the low frequency semicircle is not resolved. At 300 K, the low-frequency fit diverts from the data, presumably because of the distribution of timescales for the low-frequency regime.

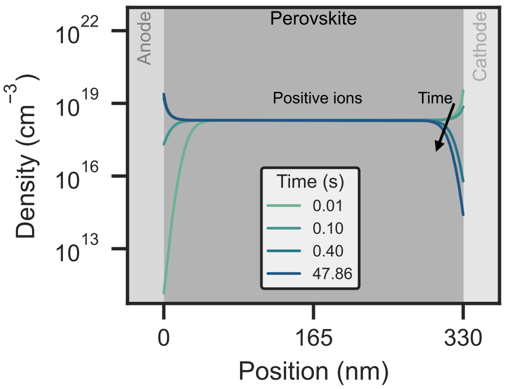

Figure S9: Simulations of mobile positive ions at 300 K within the perovskite at different times after removing a voltage pulse.

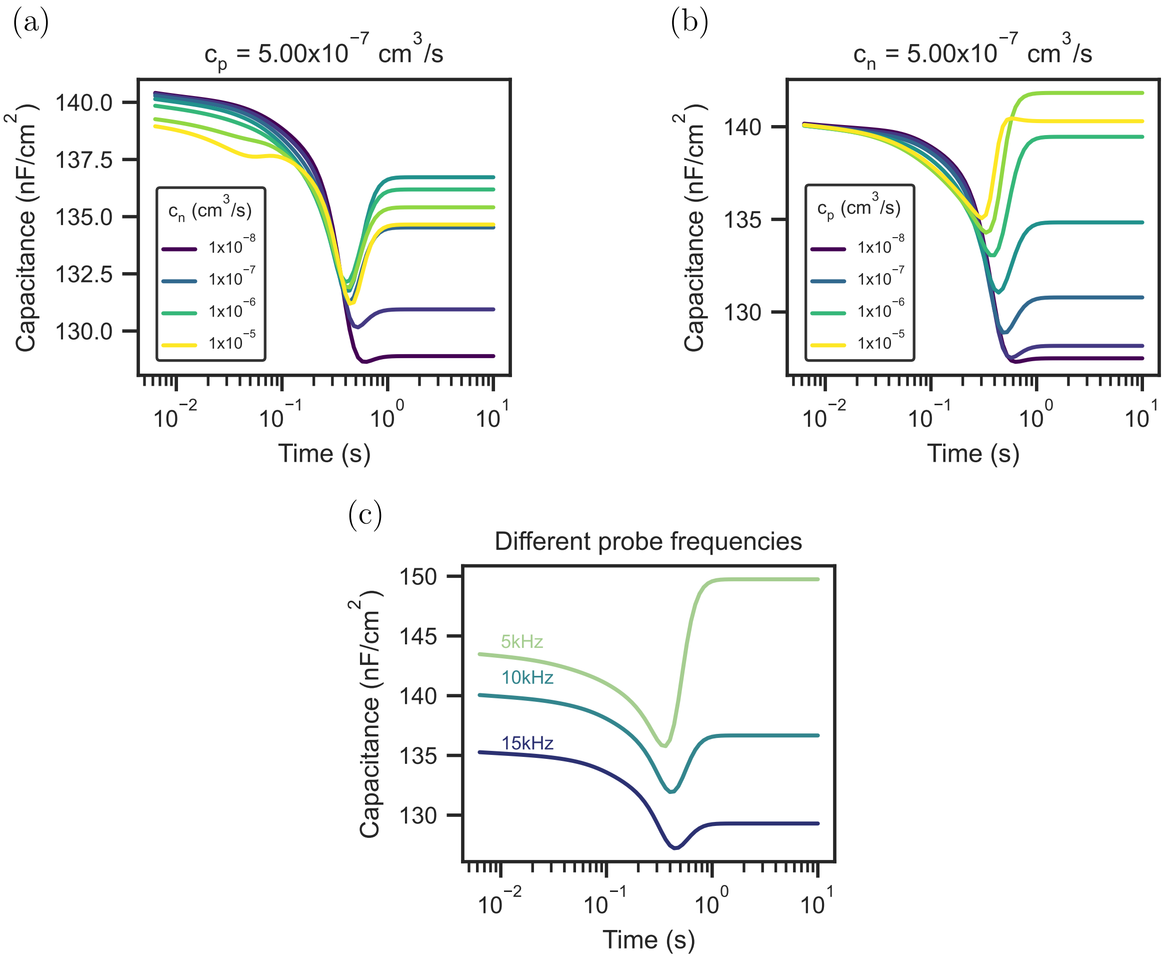

Figure S10: Simulations of capacitance transients, illustrating the dependency of the capacitance rise on the (a) electron capture rate, (b) hole capture rate, and (c) probing frequency.

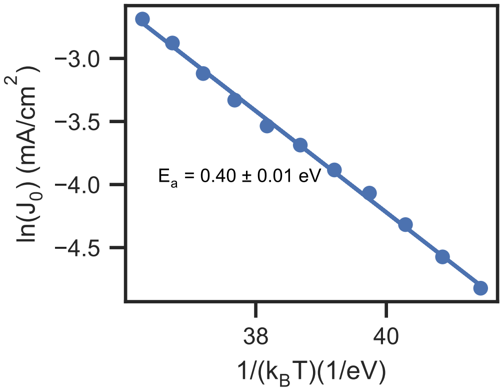

Figure S11: Extracted activation energy of the amplitude J_0 of the stretched exponential decay of the transient current measurements shown in Figure 4(a) in the main text.

References

(1) Reichert, S.; Flemming, J.; An, Q.; Vaynzof, Y.; Pietschmann, J.-F.; Deibel, C. Ionic-Defect Distribution Revealed by Improved Evaluation of Deep-Level Transient Spectroscopy on Perovskite Solar Cells. *Physical Review Applied* **2020**, *13*, 034018.

(2) Gaitan, M.; Mayergoyz, I. D. A numerical analysis for the small-signal response of the MOS capacitor. *Solid-State Electronics* **1989**, *32*, 207–213.

(3) Shockley, W.; Read, W. T. Statistics of the Recombinations of Holes and Electrons. *Physical Review* **1952**, *87*, 835–842.

(4) Caputo, M. et al. Electronic structure of MAPbI3 and MAPbCl3: importance of band alignment. *Scientific Reports* **2019**, *9*, 15159.

(5) Xia, C. Q.; Peng, J.; Poncé, S.; Patel, J. B.; Wright, A. D.; Crothers, T. W.; Uller Rothmann, M.; Borchert, J.; Milot, R. L.; Kraus, H.; Lin, Q.; Giustino, F.; Herz, L. M.; Johnston, M. B. Limits to Electrical Mobility in Lead-Halide Perovskite Semiconductors. *The Journal of Physical Chemistry Letters* **2021**, *12*, 3607–3617.

(6) Jacobs, D. A.; Shen, H.; Pfeffer, F.; Peng, J.; White, T. P.; Beck, F. J.; Catchpole, K. R. The two faces of capacitance: New interpretations for electrical impedance measurements of perovskite solar cells and their relation to hysteresis. *Journal of Applied Physics* **2018**, *124*, 225702.

(7) Guo, X.; McCleese, C.; Kolodziej, C.; Samia, A. C. S.; Zhao, Y.; Burda, C. Identification and characterization of the intermediate phase in hybrid organic–inorganic MAPbI $_3$ perovskite. *Dalton Transactions* **2016**, *45*, 3806–3813.
